# Comparative Analysis of CNV Calling Algorithms: Literature Survey and a Case Study Using Bovine High-Density SNP Data

**DOI:** 10.3390/microarrays2030171

**Published:** 2013-06-25

**Authors:** Lingyang Xu, Yali Hou, Derek M. Bickhart, Jiuzhou Song, George E. Liu

**Affiliations:** 1 Bovine Functional Genomics Laboratory, BARC, BA, USDA-ARS, Beltsville, MD 20705, USA; E-Mail: xulingyang2008@gmail.com; 2 Department of Animal and Avian Sciences, University of Maryland, College Park, MD 20742, USA; E-Mail: songj88@umd.edu; 3 Laboratory of Disease Genomics and Individualized Medicine, Beijing Institute of Genomics, Chinese Academy of Sciences, Beijing 100029, China; E-Mail: houyali1210@gmail.com; 4 Animal Improvement Programs Laboratory, BARC, BA, USDA-ARS, Beltsville, MD 20705, USA; E-Mail: Derek.Bickhart@ars.usda.gov

**Keywords:** copy number variation (CNV), algorithm, segmental duplication, single nucleotide polymorphism (SNP), cattle genome

## Abstract

Copy number variations (CNVs) are gains and losses of genomic sequence between two individuals of a species when compared to a reference genome. The data from single nucleotide polymorphism (SNP) microarrays are now routinely used for genotyping, but they also can be utilized for copy number detection. Substantial progress has been made in array design and CNV calling algorithms and at least 10 comparison studies in humans have been published to assess them. In this review, we first survey the literature on existing microarray platforms and CNV calling algorithms. We then examine a number of CNV calling tools to evaluate their impacts using bovine high-density SNP data. Large incongruities in the results from different CNV calling tools highlight the need for standardizing array data collection, quality assessment and experimental validation. Only after careful experimental design and rigorous data filtering can the impacts of CNVs on both normal phenotypic variability and disease susceptibility be fully revealed.

## 1. Introduction

Genomic structural variation, including copy number variation (CNV), has been extensively studied in humans [1,2,3,4,5] and rodents [6,7,8,9]. Initial CNV reports have also been released for domesticated animals, including dog [10,11,12], cattle [13,14], chicken [15,16], pig [17,18], sheep [19,20], and goat [21] amongst others. Recent bovine CNV studies have generated several cattle CNV maps based on the data from Illumina Bovine SNP50K microarrays [22,23,24,25].

CNVs can be identified using various approaches, including comparative genomic hybridization (CGH) arrays, SNP arrays, and DNA sequencing. In spite of the increasing adoption of next-generation sequencing, microarrays will continue to be the primary platform for CNV detection in the near future. Compared to other approaches, the advantages of SNP arrays include their relative low cost and high throughput. Substantial genotyping data have been produced from genome-wide association studies, which can be directly exploited for CNV analysis. Dozens of human and mouse CNV studies have demonstrated that some CNVs are associated with phenotypic traits and diseases [26,27,28,29]. Efforts to explore the association between cattle CNV and economical traits have been published [30,31,32], even though the actual functional mechanisms are not yet well defined.

## 2. CNV Detection Using SNP Arrays

SNP arrays were initially designed to genotype thousands of SNPs across the genome concurrently. Their applications have now expanded to include CNV detection using additional information such as the probe hybridization signal on each individual chip. The most well-known SNP microarrays are available from commercial vendors such as Illumina and Affymetrix [33,34]. Both companies sell competing arrays and continue to offer ever increasing coverage for detecting SNPs and CNVs simultaneously. However, one important consideration is the inherent bias of the SNP chip coverage against areas of the genome known to frequently harbor CNVs. For example, common copy number polymorphisms (CNPs) may cause a SNP to be rejected when the SNP fails standard inheritance checks and Hardy-Weinberg tests [35]. 

Segmental duplications (SDs), defined as >1 kb stretches of duplicated DNA with high sequence identity in a species, were shown to be one of the catalysts and hotspots for CNV formation [36,37,38]. Although the current microarray platforms offer some detection power in SD regions, calls within these regions are often affected by low probe density and cross-hybridization of repetitive sequence. In addition, only a relative copy number (CN) increase or decrease is reported with respect to the reference samples in SD regions. This poses a particular problem in the detection of CNVs in SD regions as the test individual’s copy number may differ from that of the reference by a smaller proportion than is detectable using array-based calling criteria. Although analyses of a subset of CNVs provided evidence of linkage disequilibrium with flanking SNPs [39], a significant portion of CNVs fell in genomic regions not well covered by SNP arrays, such as SD regions, and thus were not genotyped [40,41,42]. 

Since SNP chips are primarily designed for their use in SNP genotyping, some background noise that does not affect SNP calling may cause problems for CNV calling algorithms. For example, SNP data is typically normalized against a reference population in order to reduce between-array variations and probe-specific hybridization effects. The assumption that the large majority of reference samples have the same two copies does not hold for common CNV regions. At these regions, the normalization should be further optimized to derive correct parameters. Several new array designs have incorporated CNV detection, for example, monomorphic probes in common CNV regions are included on more recent Illumina and Affymetrix SNP array platforms.

## 3. Algorithms for CNV Detection

Undoubtedly, microarray development has spurred the advances in computational analysis methodology in quantitative fields of biology. A wide range of CNV discovery tools has been developed based on data derived from SNP arrays, such as cnvPartition [43], Birdsuite [44], PennCNV [45], and amongst others. In this section, we briefly introduce these CNV detection tools. 

**cnvPartition**: Illumina data can be initially viewed, processed and exported using the proprietary GenomeStudio program (Illumina, CA, USA). In addition to quality checking and genotype calling, the program calculates several important input values for CNV discovery. The log R ratio (LRR), *i.e.*, log2(R_observed_/R_expected_), is calculated from the observed normalized intensity of a sample and expected normalized intensity, which is calculated from linear interpolation of canonical genotype clusters. The B allele frequency (BAF, normalized measure of relative signal intensity ratio of the B and A alleles) is calculated from the difference between the actual value and the expected position of the cluster group. LRR and BAF are used by many CNV detection algorithms. cnvPartition is offered as a plug-in for the GenomeStudio program, where it uses LRR and BAF to assess copy number using 14 different Gaussian distribution models between zero and four copies. cnvPartition also uses a likelihood-based method to compute the confidence score for each CNV call. Given the integration of cnvPartition into Illumina proprietary software (GenomeStudio), cnvPartition is currently unable to process and analyze Affymetrix chip data.

**Birdsuite**: Affymetrix SNP array data from older chips must first be analyzed in the Genotyping Console program provided by Affymetrix for initial quality checks and controls. Data from the newer Affymetrix chip can be processed by additional programs contained in the Birdsuite package [44]. The Canary module of Birdsuite genotypes the known common CNVs using an Expectation-Maximization (EM) algorithm while the Birdseye module detects novel CNVs by using a Hidden Markov Model (HMM) with a Viterbi algorithm calculating emission states. For Affymetrix SNP arrays, there are other freely available CNV detection programs, such as GADA [46], Cokgen [47], iPattern [26] in addition to Birdsuite. For details about these programs, please see these published reviews [35,48,49]. The developers of Birdsuite have mentioned future plans for Illumina platform support [50] but current options only include a beta version for Illumina 610 array platforms. 

**PennCNV and QuantiSNP**: PennCNV and QuantiSNP are two freely available programs developed based on HMMs [45,51]. Both programs can process Illumina and Affymetrix SNP data. PennCNV incorporates multiple sources of information, including LRR and BAF at each SNP marker, the distance between neighboring SNPs and the allele frequency of SNPs. PennCNV also integrates a computational approach by fitting regression models with GC content to overcome “genomic waves” [52,53]. Additionally, PennCNV is capable of considering pedigree information (a parents-offspring trio) to improve call rates and accuracy of breakpoint prediction as well as to infer chromosome-specific SNP genotypes in CNVs. Finally, PennCNV also reports data quality control measurements for each CNV dataset. 

QuantiSNP, by contrast, uses an Objective Bayes approach [51] to infer copy number states based on the LogR ratio and the B allele frequency for each SNP marker. Whereas the PennCNV algorithm uses a transition matrix to model realistic copy number transitions between SNP probes [45], QuantiSNP calculates Bayesian probabilities for each SNP marker pair and then uses a HMM to join markers to form CNVs. Another significant difference between the two programs is that PennCNV is an open-source project whereas QuantiSNP was written for MatLab, which may limit availability to users that may not have a MatLab license. Finally, QuantiSNP is no longer under active development as listed on its webpage [54].

**Approaches originally developed for array CGH**: Several tools for CNV detection, which were originally developed for array CGH CNV calling, have been modified for SNP array analysis. However, these methods normally do not consider BAF information, which is the preferred data source to use for CNV calling in SNP data. For example, the Circular Binary Segmentation (CBS) method was designed to convert noisy intensity values into neighboring segments of distinct assigned copy numbers using dynamic programming [55]. DNAcopy is a widely used R implementation of the CBS method.

**Other commercial CNV detection tools**: Other commercially available programs include Partek Genomics Suite, Nexus Copy Number software and Golden Helix SNP & Variation Suite (SVS). The strength of these commercial tools include their graphical user interfaces, streamlined pipelines for analysis and work flow, optimized computational speed as well as technical support. These factors are very important to labs with limited bioinformatics support; however, commercial companies often do not utilize some of the latest methods developed in the academic environment. For this study, we have chosen to look in detail at the Golden Helix SVS [56]. The SVS Copy Number Analysis Module (CNAM) employs a segmentation algorithm using only the signal intensity data to detect CNVs on either a per-sample (univariate) or multi-sample (multivariate) basis. According to its online manual, the univariate method, which considers only one sample at a time, is designed for detecting rare and/or large CNVs. The multivariate method, which considers all samples simultaneously, is designed for detecting small, common CNVs. 

**Comparing univariate and multivariate methods**: Although the exact algorithm of each method is proprietary, Breheny *et al.* explored the strengths and weaknesses of two similar approaches using both simulations and real data [57]. In their study, the univariate method (the CNV-level testing, *i.e.*, across markers within one sample) involves estimating, at the level of the individual genome, the underlying copy number at each location. Once this is completed, tests are performed to determine the association between copy number state and phenotype. The multivariate method (the pooled marker-level testing across samples) carries out association testing first between the phenotypes and raw intensities at the level of the individual marker, and then aggregates neighboring test results to identify CNVs associated with the phenotype. Accounting for multiple comparisons across SNP markers is more straightforward, as a multiple-comparison correction (e.g., Bonferroni, permutation) can directly control the family-wise error rate (FWER) of the overall procedure [58]. False discovery rates can be calculated to account for multiple comparisons with the CNV-level testing method [59]; however, this is more complicated and somewhat conservative. Partially overlapping CNVs across cell lines introduce dependence across the tests, thereby reducing the effective number of independent tests. Breheny *et al.* confirmed that that the univariate method/CNV-level testing has greater power to detect associations involving large, rare CNVs, while the multivariate method/pooled marker-level testing has greater power to detect associations involving small, common CNVs. It is important to understand these tradeoffs. Several recent papers have proposed to develop methods capable of simultaneously pooling information across both markers and samples for CNV detection and association studies [60,61,62,63,64].

**CNV quality score**: Many programs like cnvPartition, Birdsuite, PennCNV and QuantiSNP reported CNV quality scores, which are quantitative values indicating CNV confidences. Although their exact meanings and interpretations depend on each algorithm and they are often not reported in microarray studies. These CNV quality scores are important for constructing CNV regions, which can then be used in association studies.

## 4. Comparing the CNV Detection Algorithms Using Human Data

As shown in Table 1, at least 10 comparisons of the strengths and weaknesses of these array platforms and CNV calling tools have been published using human CNV data. Although published results are quickly outdated as new platforms and tools are introduced, a general theme is consistent across these comparisons. The first of these is the lack of a standard approach to collecting the data and the lack of standardized reference samples; this makes it difficult to compare CNV results across different studies [65]. The second is that CNV results also differ substantially depending on CNV detection methods [35,49]. For example, as the most comprehensive study on this topic, Pinto *et al.* have systematically compared CNV detection on 11 microarray platforms to evaluate data quality and CNV calling, reproducibility, concordance across array platforms and laboratories, breakpoint accuracy and analysis tool variability [49]. It is surprising that reproducibility in replicate experiments is <70% for most platforms and different analytic tools applied to the same raw data typically yield CNV calls with <50% concordance. The authors attributed these poor reproducibility observations to these facts: (1) large CNVs often overlap with SDs in complex genomic regions (as we described before) and (2) large CNVs also lead to call fragmentation (a single CNV is detected as multiple smaller variants). This led the authors to conclude that, “the striking differences between CNV calls from different platforms and analytic tools highlight the importance of careful assessment of experimental design in discovery and association studies and of strict data curation and filtering in diagnostics” [49].

**Table 1:** Survey of recent comparison studies of copy number variation (CNV) detection.

Authors	Year	Algorithm	Data	Platform		Vendor	Conclusion	Comment
Lai [66]	2005	CGHseq, Quantreg, CLAC, GLAD, CBS, HMM, Wavelet, Lowess, ChARM, GA and ACE	Simulation and empirical samples for Glioblastoma	array CGH		Custom cDNA array	Several general characteristics of future program development were suggested.	Earlier programs for array CGH.
Baross [67]	2007	CNAG, dChip, CNAT, GLAD	Simulation and empirical mental retardation 100K Affymetrix SNP array	SNP array		Affymetrix	Multiple programs were needed to find all real aberrations.	False positive deletions was substantial, but could be greatly reduced by using the SNP genotype information to confirm loss of heterozygosity.
Winchester [35]	2009	Birdsuite, CNAT, GADA, PennCNV, QuantiSNP	NA12156, NA15510	SNP array		Affymetrix, Illumina	Multiple predictions from different software.	Use software designed for the platform.
Dellinger [68]	2010	CBS, cnvFinder, cnvPartition, GALD, Nexus, PennCNV and QuantiSNP	Simulation and empirical samples from Singapore cohort study of the risk factors for Myopia	SNP array		Illumina	QuantiSNP outperformed other methods based on ROC curve residuals over most datasets. Nexus Rank and SNPRank have low specificity and high power. Nexus Rank calls oversized CNVs. PennCNV detects one of the fewest numbers of CNVs.	The normalized singleton ratio (NSR) is proposed as a metric for parameter optimization.
Tsuang [69]	2010	PennCNV, QuantiSNP, HMMSeg, and cnvPartition	48 Schizophrenia samples	SNP array		Illumina	Both guidelines for the identification of CNVs inferred from high-density arrays and the establishment of a gold standard for validation of CNVs are needed.	Given the variety of methods used, there will be many false positives and false negatives.
Zhang [70]	2011	Birdsuite, Partek Genomics Suite, HelixTree, and PennCNV-affy	~1,000 Bipolar + 270 HapMap samples	SNP array	Affymetrix		Birdsuite and Partek had higher positive predictive values.	Poor overlap between 2 gold standards (Kidd et al. and Conrad et al.).
Marenne [71]	2011	cnvPartition, PennCNV, and QuantiSNP	96 pair samples from Spanish Bladder Cancer/EPICURO study	SNP array	Illumina		PennCNV was the most reliable algorithm when assessing the number of copies.	Current calling algorithms should be improved for high performance CNV analysis in genome-wide scans.
Pinto [49]	2011	Birdsuite, cnvFinder, cnvPartition, dCHIP, ADM-2 (DNA Analytics), Genotyping Console (GTC), iPattern, Nexus Copy Number, Partek Genomics Suite, PennCNV, QuantiSNP	6 samples in triplicate on 11 array platforms	array CGH, SNP array, and BAC array	Agilent, NimbleGen, Affymetrix, and Illumina		Different analytic tools applied to the same raw data typically yield CNV calls with <50% concordance. Moreover, reproducibility in replicate experiments is <70% for most platforms.	The CNV resource presented here allows independent data evaluation and provides a means to benchmark new algorithms. CNV calls are disproportionally affected by genome complexity as they tend to overlap SDs and a single CNV is detected as multiple smaller variants.
Koike [48]	2011	Birdsuite, Birdseye, PennCNV, CGHseg, DNAcopy	HapMap samples	SNP array	Affymetrix		Hidden Markov model-based programs PennCNV and Birdseye (part of Birdsuite), or Birdsuite show better detection performance.	Segmental duplications and interspersed repeats (LINEs) are involved in CNVs.
Eckel-Passow [72]	2011	Affymetrix Power Tools (APT), Aroma.Affymetrix, PennCNV and CRLMM	1,418 GENOA (Genetic Epidemiology Network of Atherosclerosis)/FBPP (Family Blood Pressure Program) samples	SNP array	Affymetrix		Recommended trying multiple algorithms, evaluating concordance/discordance and subsequently consider the union of regions for downstream association tests.	Advocated that software developers need to provide guidance with respect to evaluating and choosing optimal settings in order to obtain optimal results for an individual dataset.

## 5. Comparing CNV Detection Algorithms Using Bovine High-Density SNP Data

We performed an analysis of CNVs based on Illumina BovineHD chips, which contain more than 750,000 SNP markers [73], using PennCNV. As a consequence of the higher SNP count, more CNVs were identified with higher resolution boundaries. In order to provide an additional comparison of CNV detection methods, we have tested three additional tools to call CNVs on the same BovineHD dataset: cnvPartition version 3.6.1, Golden Helix SVS 7.0 and DNAcopy [55]. These four tools were applicable to our dataset (Illumina bead array), available to us (due to existing commercial licensing or free availability) and were not designed specifically for human-based array studies. 

In order to perform an accurate and fair comparison of calls across the different methods, our PennCNV calls were derived from the same 630 animals of 27 cattle breeds on the cattle reference assembly UMD3.1 without using trio information [73]. We carried out cnvPartion calling using the default parameters as recommended by Illumina. For the Golden Helix SVS7.0, we used the SVS DSF Export Plug-In 4.1 to export LRRs from the GenomeStudio project. We then utilized CNAM to process the DSF file under the univariate option (minimum 3 markers/segment, a significance level of *p* = 0.005 for 2,000 pairwise permutations). We also performed DNAcopy analysis based on LRR. Finally, CNV segments were then filtered with a minimum of 3 probes for all 4 tools and a minimum of absolute segment mean values of 0.3 for SVS and DNAcopy.

**Table 2:** CNVs and CNVRs identified using PennCNV, cnvPartition, SVS, and DNAcopy.

Tool	Event	Count	Gain	Loss	Average Length
**PennCNV**	CNV	46,751 (74.2)	17,796 (28.2)	28,955 (46.0)	2,334,244,479 (49,929)
	CNVR	3,364^ a^	1,382^ b^	2,376^ c^	147,476,461 (43,840)
**cnvPartition**	CNV	16,566 (26.3)	5,021 (8.0)	11,545 (18.3)	2,191,528,246 (132,291)
	CNVR	1,298^ a^	541^ b^	916^ c^	172,378,730 (132,803)
**SVS**	CNV	92,463 (146.8)	205 (0.3)	92,258 (146.4)	2,234,601,290 (24,168)
	CNVR	7,099^ a^	78^ b^	7,056^ c^	151,471,634 (21,337)
**DNAcopy**	CNV	41,858 (66.4)	4,469 (7.1)	37,389 (59.3)	1,863,930,368 (44,530)
	CNVR	5,961^ a^	1,457^ b^	5,284^ c^	194,287,154 (32,593)

Numbers in parentheses are values normalized by sample counts, except in the case of the parentheses values in the “Average Length” column, which are average lengths normalized by CNV counts. ^a^ These numbers represent non-redundant CNVR counts after merging both gain and loss CNVs identified across all 630 samples. ^b^ Gain CNV events were merged separately. ^c^ Loss CNV events were merged separately.

**Figure 1:**
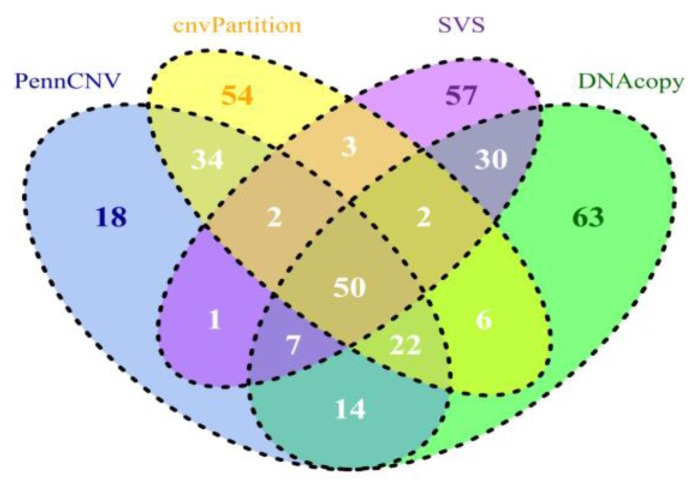
Comparisons of CNVR results identified by PennCNV, cnvPartition, SVS, and DNAcopy based on genomic location in UMD3.1. The overlap lengths of CNVRs were indicated in Mb.

A summary of CNV and CNVR results derived from all 630 samples is shown in Table 2. Detailed results can be found in the four worksheets of Supplementary Table 1. Compared to PennCNV results, CNVs and CNVRs in cnvPartition results are fewer and ~3 times longer (45 kb *vs.* 130 kb, respectively). While PennCNV and cnvPartition have loss/gain ratios of ~1.7 and DNAcopy has a ratio of 3.6, SVS has a ratio over 90, suggesting SVS is more sensitive to loss events than to gain events. Additionally, both SVS and DNAcopy CNVRs (average length approximately 20 kb and 30 kb, respectively) are shorter than PennCNV (~40 kb), and significantly shorter than cnvPartition CNVRs (~132 kb). Similar observations were also obtained when each subspecies/group (*i.e.* taurine, indicine, composite (taurine × indicine) and African breeds) was processed separately, confirming the above results (data not shown). When we compared CNV calls across subspecies/groups for all four CNV calling methods, CNV counts per sample were higher in African and indicine breeds, intermediate in composite breeds, and lower in taurine breeds, agreeing with our previous results using PennCNV [73]. We then compared the CNVRs from the four datasets derived from our calling programs based on the UMD3.1 cattle reference assembly (Figure 1). Approximately 50 Mb of core CNVRs are shared among the four CNVR sets. We calculated concordances using the ratios of between intersections and unions for both counts and lengths (Table 3 and Figure 1). PennCNV shared more regions (108 Mb or 50.82%) by length and 43.80% by count with cnvPartition than with any other tools. Therefore, we have observed that tools based on similar algorithms and input data (both LRR and BAF) seem to share more common regions. By contrast, PennCNV and SVS shared 24.88% or 60 Mb in length and 13.74% by count. This comparison was consistent with a recent publication based on PennCNV and SVS using human autism samples [74]. When we applied different filtering criteria requiring a minimum of five or 12 probes, the overlap of calls from these two methods increased slightly, ranging from 24–52% by the number of nucleotides that overlapped. We also evaluated the overlaps between loss CNV events across four datasets for each individual sample. The number of bases overlapped by CNVs from each dataset ranged from 26–48%, which agreed with our CNVR overlapping results.

**Table 3:** Overlaps among CNVRs across 4 CNV detection tools.

		Count			Length (base pair)		
Tool1	Tool2	Intersection ^a^	Union^ a^	Percentage	Intersection^ b^	Union^ b^	Percentage
PennCNV	cnvPartition	1,420	3,242	43.80%	107,775,740	212,079,451	50.82%
PennCNV	DNAcopy	2,355	6,970	33.79%	93,149,061	248,614,554	37.47%
PennCNV	SVS	1,264	9,199	13.74%	59,557,597	239,390,498	24.88%
cnvPartition	DNAcopy	1,284	5,975	21.49%	79,825,624	286,840,260	27.83%
cnvPartition	SVS	981	7,416	13.23%	56,569,347	267,281,017	21.16%
DNAcopy	SVS	2,332	10,728	21.74%	88,864,805	256,893,983	34.59%

^a^ These numbers represent intersections and unions of two CNVR datasets by count. ^b^ These numbers represent intersections and unions of two CNVR datasets by length in base pair.

## 6. Conclusions

Like other published comparisons of CNV calling methods, we observed large variations in calls made by different programs. As pointed out previously, hybridization studies will generate both false positive and false negative results, regardless of how the data are analyzed [75]. As shown in Table 1, many authors recommended using multiple CNV calling algorithms instead of just one [35]; however, although the net effect of this strategy decreases the false negative rate, it also increases the false positive rate. With next generation sequencing projects producing better CNV calling standards, such as the 1,000 human genomes project [5] and our recent effort [76], we should be able to better estimate the false positive and false negative rates for each tool. Large incongruities in the results from different CNV calling tools highlight the need for standardizing array data collection, quality assessment and experimental validation. This is extremely true for other species like cattle, for which there is no gold standard of CNV calls to compare data against. Only after careful experimental design and rigorous data filtering can the impacts of CNVs on both normal phenotypic variability and disease susceptibility be fully revealed.
